# Decoding temporal heterogeneity in NSCLC through machine learning and prognostic model construction

**DOI:** 10.1186/s12957-024-03435-0

**Published:** 2024-06-13

**Authors:** Junpeng Cheng, Meizhu Xiao, Qingkang Meng, Min Zhang, Denan Zhang, Lei Liu, Qing Jin, Zhijin Fu, Yanjiao Li, Xiujie Chen, Hongbo Xie

**Affiliations:** Department of Pharmacogenomics, College of Bioinformatics Science and Technology, Harbin Medical University, Harbin, 150086 P. R. China

**Keywords:** Non-small cell lung cancer, Temporal heterogeneity, Machine learning

## Abstract

**Background:**

Non-small cell lung cancer (NSCLC) is a prevalent and heterogeneous disease with significant genomic variations between the early and advanced stages. The identification of key genes and pathways driving NSCLC tumor progression is critical for improving the diagnosis and treatment outcomes of this disease.

**Methods:**

In this study, we conducted single-cell transcriptome analysis on 93,406 cells from 22 NSCLC patients to characterize malignant NSCLC cancer cells. Utilizing cNMF, we classified these cells into distinct modules, thus identifying the diverse molecular profiles within NSCLC. Through pseudotime analysis, we delineated temporal gene expression changes during NSCLC evolution, thus demonstrating genes associated with disease progression. Using the XGBoost model, we assessed the significance of these genes in the pseudotime trajectory. Our findings were validated by using transcriptome sequencing data from The Cancer Genome Atlas (TCGA), supplemented via LASSO regression to refine the selection of characteristic genes. Subsequently, we established a risk score model based on these genes, thus providing a potential tool for cancer risk assessment and personalized treatment strategies.

**Results:**

We used cNMF to classify malignant NSCLC cells into three functional modules, including the metabolic reprogramming module, cell cycle module, and cell stemness module, which can be used for the functional classification of malignant tumor cells in NSCLC. These findings also indicate that metabolism, the cell cycle, and tumor stemness play important driving roles in the malignant evolution of NSCLC. We integrated cNMF and XGBoost to select marker genes that are indicative of both early and advanced NSCLC stages. The expression of genes such as *CHCHD2*, *GAPDH*, and *CD24* was strongly correlated with the malignant evolution of NSCLC at the single-cell data level. These genes have been validated via histological data. The risk score model that we established (represented by eight genes) was ultimately validated with GEO data.

**Conclusion:**

In summary, our study contributes to the identification of temporal heterogeneous biomarkers in NSCLC, thus offering insights into disease progression mechanisms and potential therapeutic targets. The developed workflow demonstrates promise for future applications in clinical practice.

**Supplementary Information:**

The online version contains supplementary material available at 10.1186/s12957-024-03435-0.

## Introduction

Non-small cell lung cancer (NSCLC) is the primary pathological type of lung cancer, accounting for more than 80% of all lung cancer cases; moreover, its development is a complex process involving genetic and environmental factors [1, 2]. Early detection and treatment of NSCLC are critical factors that significantly improve patient outcomes. More importantly, NSCLC is a disease with high temporal heterogeneity. The genomes of early-stage and advanced-stage NSCLC patients exhibit notable variations [3, 4]. These variations ultimately contribute to alterations in the tumor microenvironment, the metastasis of cancer cells, and the emergence of drug resistance.

In recent years, extensive research has focused on elucidating heterogeneously derived mechanisms in NSCLC patients and understanding their implications for clinical practice. *EGFR*, *KRAS*, *ALK*, and *TP53* are commonly mutated in NSCLC and play crucial roles in its progression [5, 6]. For example, genetic mutations (such as those in *EGFR*) can affect patient prognosis and responses to targeted therapies [7–9]. Chabon et al. utilized CAPP-Seq ctDNA analysis to examine resistance mechanisms in 43 NSCLC patients treated with a third-generation *EGFR* inhibitor. Following treatment with first-line inhibitors, 46% of the patients exhibited multiple resistance mechanisms, thus indicating significant intrapatient heterogeneity [10]. *KRAS* has a high mutation rate, particularly in lung cancer, wherein it occurs in 31–35% of patients. Point mutations are prevalent in the *KRAS* gene, thus leading to a constantly active GTP-bound state and activation of downstream oncogenic pathways. Despite extensive preclinical and clinical research, there are currently no approved therapies specifically targeting mutated *KRAS* or its downstream signaling pathways. Alterations in *ALK* and *TP53* also warrant attention in the diagnosis and treatment of NSCLC [11]. Briefly, the emergence of heterogeneity in NSCLC is a complex process involving many gene alterations. Therefore, the accurate prediction of the progression of NSCLC and the identification of genes that can affect the progression of NSCLC are highly important for the diagnosis and treatment of this disease.

The tumor microenvironment (TME), which encompasses various cell types, stromal elements, and immune cells, also plays a crucial role in NSCLC heterogeneity. Interactions within the tumor microenvironment can influence treatment responses and the development of resistance to therapy, thus highlighting the importance of considering tumor-host interactions in clinical practice. Cords et al. utilized single-cell imaging mass cytometry to analyze cancer-associated fibroblasts (CAFs) in 1,070 NSCLC patients and identified four prognostic groups based on 11 phenotypes. The presence of tumor-like CAFs is correlated with poor prognosis, whereas the presence of inflammatory and interferon-responsive CAFs predicts better outcomes. A high matrix CAF density is correlated with low immune infiltration and reduced survival. These findings emphasize the need for therapies targeting CAFs with poor prognosis or supporting those associated with favorable outcomes [12]. Using single-cell RNA sequencing (scRNA-seq), Wu et al. described the landscape of immune cells, stromal cells, and cancer cells in advanced NSCLC. These authors found that neutrophils were enriched in LUSC, which is consistent with previous studies showing higher neutrophil levels in LUSC than in LUAD due to variations in the TME. However, LUAD patients exhibited stronger cancer-neutrophil interactions. These findings suggest diverse roles for neutrophils in the TME, thus highlighting their potential impacts on immunotherapy efficacy [13]. Moreover, epigenetic alterations contribute to the heterogeneity of NSCLC by modulating gene expression patterns. An understanding of the epigenetic landscape of lung tumors may offer insights into novel therapeutic targets and predictive biomarkers [14, 15]. In summary, NSCLC is significantly associated with tumor heterogeneity. An understanding of the temporal dynamics of NSCLC can provide valuable insights into the potential mechanisms driving tumor evolution, drug resistance, and disease recurrence.

The prediction of the development and evolution of cancer is a feasible and essential area of research. In addition to pathological staging, other tools, such as genetic testing and imaging, can be used to predict cancer progression. Advances in technology and data analysis are also making it possible to predict cancer progression with increasing accuracy. Machine learning algorithms have also shown outstanding performance in discovering gene expression patterns, identifying biomarkers, and annotating the genome. Moreover, substantial advancements in single-cell sequencing technology have significantly contributed to the extensive investigation of tumor heterogeneity and evolution in recent research [16, 17]. In a study by Wu et al. using scRNA-seq and spatial transcriptomics data from 24 patients, the authors explored the immune atlas of colorectal cancer liver metastasis. They demonstrated that the immune microenvironment underwent dynamic cellular and spatial changes from the primary tumor to the metastatic microenvironment [18].

**Fig. 1:**
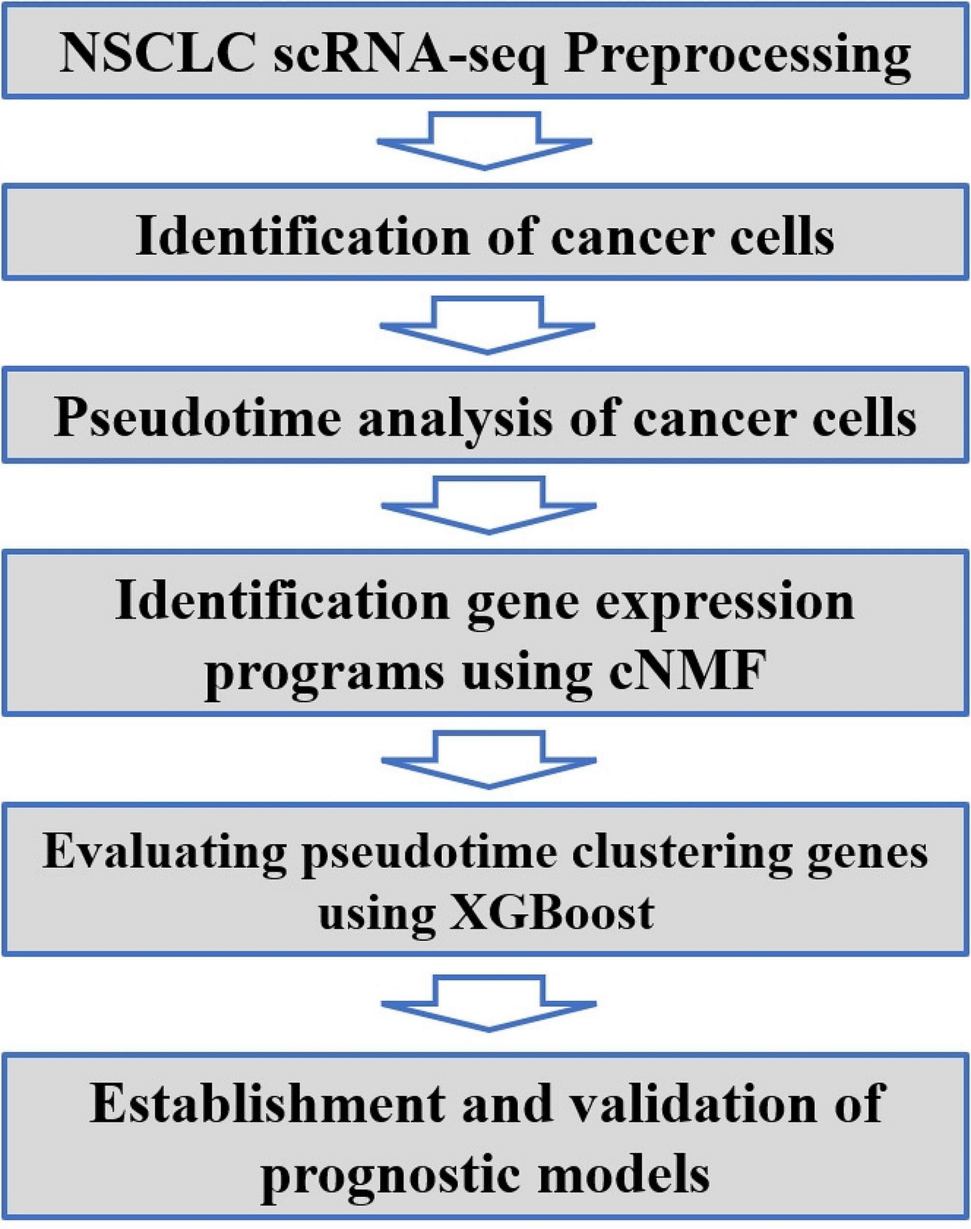
The flowchart for this study

The aim of this study was to integrate single-cell transcriptome and bulk transcriptome data by employing various machine learning algorithms to discern gene markers distinguishing between early-stage and advanced-stage of NSCLC tumors. Concurrently, we investigated the underlying biological mechanisms that contribute to the development of NSCLC. In the analysis of the scRNA-seq data, we conducted consensus nonnegative matrix factorization (cNMF) on the identified NSCLC cells, thus resulting in a total of 12 expression programs. Through enrichment analysis of the top 50 genes with the highest weight in each expression program, we identified relevant pathways. Pathways such as metabolic reprogramming, the cell cycle, and cell stemness pathways exhibited increased activity in NSCLC. We examined variations in the composition of single-cell transcriptomes between early-stage and advanced-stage NSCLC. Utilizing pseudotime analysis, we identified 2037 genes associated with cancer progression. Subsequently, we utilized XGBoost to assess the significance of these genes in the temporal development of cancer cells. We then employed these overlapping selected genes to develop a LASSO regression model using the TCGA training set. Afterwards, we validated the accuracy of the model by using GEO data. The workflow of this study is illustrated in Fig. 1.

## Materials and methods

### Data sources

The scRNA-seq data from 22 NSCLC patients were obtained from the Gene Expression Omnibus (GEO) database (GSE131907, GSE127465, and GSE136246). The information of the 22 patients is listed in Supplementary Table S1. We obtained a bulk transcriptome dataset and survival data from The Cancer Genome Atlas (TCGA) and the Gene Expression Omnibus database (GSE30219).

### Identification of major cell types

The single-cell gene expression matrices were converted to Seurat objects via the R package [19]. The proportions of gene numbers, cell counts, and mitochondrial sequencing counts were then computed. Only genes that were observed in at least three cells were retained. Cells with fewer than 200 or more than 5000 identified genes, as well as those with a high mitochondrial concentration (> 30%), were removed. A total of 93,406 cells were kept for further investigation after low-quality cells were discarded. We normalized expression matrices and corrected variation regression for factors. Subsequently, we identified the top 2000 variable genes and performed principal component analysis. We used the Harmony package to debatch the data. We used the first 15 principal components to compute the k.param nearest neighbors and identified 7 cell clusters.

Based on the normalized expression of the following canonical markers, we identified cell types and ultimately distinguished 7 major cell types. All of the utilized gene markers are displayed in Supplementary Figure S1.

### InferCNV analysis

InferCNV offers a robust solution for detecting CNV events at the single-cell level, thereby enabling the identification of genomic alterations and heterogeneity within cellular populations [20, 21]. The utilization of InferCNV in our study is grounded in its efficacy as a tool designed for single-cell analysis, particularly for inferring CNVs from scRNA-seq data. This capability is pivotal for identifying genetic mechanisms underlying disease progression. Overall, the adoption of InferCNV aligns with our objective of comprehensively dissecting the genetic architecture of cellular populations and elucidating its implications in disease pathogenesis. For the InferCNV analysis (version 1.14.2), we used nonmalignant cells including endothelial cells as baselines to estimate the CNV of malignant cells. Briefly, genes were sorted by their genomic locations on each chromosome. We utilized the default Hidden Markov Model (HMM) cutoff set to 0.1 for the denoising step. We filtered out CNVs with low probability by using a default threshold of 0.5. Afterwards, we used GRCh38 chromosome fragment information and annotated it as either increased or absent. Overall, our approach allowed us to accurately infer large-scale CNVs from scRNA-seq data and distinguish malignant cells from normal cells. To identify CNVs, we used endothelial cells as the reference group and epithelial cells as the observation group.

### Trajectory analysis

We applied the Monocle2 (2.24.0) package to identify lineage differentiation of cell subtypes with potential developmental relationships [22]. Monocle is a widely used software tool for analyzing single-cell data, particularly for reconstructing developmental trajectories or pseudotime ordering. Monocle utilizes various algorithms to infer cellular trajectories and identify genes that drive cell fate decisions during development or other biological processes. It is valuable for studying dynamic processes such as cell differentiation, development, and disease progression at the single-cell level [23–25]. To track the evolutionary trajectory of LUAD tumors, we employed AT2 cells and LUAD cancer cell clusters for both normal epithelial cells and cancer cells [26]. The “celldataset” object was created by using the single-cell expression matrix, and the size and dispersion of the data were assessed. We identified cluster-specific DEGs with a q value < 0.01 to determine cell differentiation. After reducing the dimensionality, the cells are ordered in a pseudotime sequence to obtain a cell trajectory. Subsequently, we identified pseudotime-dependent genes expressed in more than 1000 cells with a q value < 0.01. We visualized these genes and clustered them based on their expression patterns by using a heatmap.

### Consensus NMF molecular subtype construction

In this study, we employed Consensus Nonnegative Matrix Factorization (cNMF) (version 1.4), which is a method that is specifically designed for the analysis of scRNA-seq data, to infer the activity program of tumor cells [27, 28]. Single-cell data often exhibit challenges due to their high dimensionality and sparsity, thus rendering their analysis highly intricate. cNMF is notable as being a robust method for both dimensionality reduction and feature extraction, thus offering an effective solution for single-cell data analysis. Primarily, cNMF accomplishes the transformation of high-dimensional data into a lower-dimensional representation through matrix factorization, thereby streamlining the data structure for subsequent analysis. Second, the nonnegativity constraint inherent in cNMF ensures that the decomposed matrix retains clear biological significance, such as gene and cell features, thus facilitating the extraction of crucial information from the data and identifying intercellular heterogeneity. Third, given the prevalence of zero or near-zero values in single-cell data, which is attributed to the sparsity of gene expression data for individual cells, cNMF adeptly addresses this sparsity issue, thereby preserving essential information within the dataset. Our analysis of NSCLC tumor cells adhered to the workflow outlined by cNMF. We initiated the process with the preprocessing of raw count data, followed by matrix factorization to identify distinctive gene expression patterns. To mitigate potential batch effects that could impact the integrity of our analysis, we conducted cNMF analysis on two distinct datasets separately. The determination of the optimal number of components, which is denoted as K, played a pivotal role. We relied on assessing the model’s stability and reconstruction error by using K-selection plots to identify the most suitable value for K. After determining a suitable K value, we performed consensus analysis on the expression programs for each sample to affirm patterns that are consistently present across multiple NMF decompositions. Furthermore, we filtered for core expression programs that are extensively expressed across the cell population by setting a threshold for usage frequency (0.1), thus ensuring biological significance and reliability in our data.

### Functional and pathway enrichment analysis

Using the enrichGO function of the clusterProfiler package, the reference genome was called through the org.Hs.eg.db package, and GO terms with *p* values and q values less than 0.05 were obtained via GO enrichment analysis of the pseudotime-dependent genes that were obtained in the previous step. The R programs “digest” and “GOplot” were used to perform the GO enrichment analysis. The names of the marker genes were transferred to gene IDs. Significant enrichment was set as a *p* value < 0.05.

### Evaluating the importance of genes with pseudotime clustering using XGBoost

To further identify genes associated with the development of NSCLC, we utilized the XGBoost R package to screen for marker genes that distinguish between early-stage NSCLC and advanced-stage NSCLC. In selecting XGBoost as the primary tool for our study, we aimed to leverage its well-documented effectiveness in handling complex datasets and addressing classification tasks. XGBoost is renowned for its scalability and efficiency, particularly when considering large volumes of data. Given the diverse nature of cancer cell datasets, which are characterized by numerous features and variables, we sought a tool that could accommodate such complexity without compromising computational performance. Many previous studies have successfully utilized XGBoost for feature gene selection, thus demonstrating its efficacy [29–31]. Therefore, our application of this method to screen for key genes involved in the temporal heterogeneity of NSCLC is justified. This screening was based on gene clustering results obtained from pseudotime analysis. All of the identified cancer cells, which are categorized into early and advanced stages based on patient source, were randomly divided into a training set (70%) and a validation set (30%). This division allows us to train our predictive model on a substantial portion of the data while retaining a separate subset for evaluating its performance. By iteratively adjusting these parameters based on the performance metrics observed on the training set, we aimed to develop a model that effectively captures the underlying patterns in the data. Subsequently, the validation set plays a crucial role in assessing the generalizability of our model. By evaluating how well the model performs on data that were not used for training, we can assess its capacity to accurately classify cancer cells across various subsets of the dataset. This step helps in mitigating the risk of overfitting, wherein the model performs well on the training data but fails to generalize to unseen data. In our study, we employed the XGBoost feature importance score derived from the training set to aid in the selection of genes during the screening process. By identifying the most informative features, our model can prioritize genetic markers associated with cancer progression, thus enhancing the reliability of our findings. Overall, the inclusion of both training and validation sets strengthens the credibility of our study findings by ensuring the robustness and generalizability of our predictive model. We configured the XGBoost model with a learning rate of 0.01 and introduced a gamma value of 0.2. The maximum tree depth was constrained to 6, and the minimum child weight was set to 3. The XGBoost feature importance score was used to aid in the selection of genes during the screening process.

### The human protein atlas

The immunohistochemistry data of *CHCHD2*, *CEACAM5*, *GAPDH* and *CD24* expression in NSCLC and corresponding normal tissues were also retrieved from the Human Protein Atlas (HPA) database [32].

### Construction of the risk score

The overlap between genes identified through cNMF and genes selected by XGBoost was utilized to construct the risk score. LASSO regularized regression was performed to select for smaller features that were most strongly associated with OS in patients in the TCGA-LUAD cohort. Cox regression analysis was also used for gene set selection. We conducted LASSO regression by using the R “glmnet” package, and the genes that were screened out were utilized to establish a prognostic model. Specifically, gene names of interest were chosen as the inputs to generate survival curves for overall patient survival. We used “median” as the group cutoff metric to assign the lower and higher halves of the patients to the low and high groups, respectively. The log-rank test was used to determine whether there were significant differences in the survival distributions between the two groups. A *p* value < 0.05 was statistically significant. Patients were dichotomized into a high-risk group and a low-risk group by using the median risk score and subsequently analyzed for differences in overall survival by using the R “survival” package. The dataset from GSE30219 (*n* = 307) was used to test the model.

### Cell–cell interaction prediction in single-cell transcriptomic data

To explore the potential cell interactome in our data, we used the CellChat package (version 1.6.1) to predict interactions between different cell types based on single-cell RNA sequencing data [33]. By utilizing network analysis and pattern recognition techniques, CellChat predicts the primary signal inputs and outputs of cells, thus elucidating the coordination of cell functions and signal interactions. It has been widely applied in the field of cell‒cell interactions [34–36]. This package infers enriched receptor‒ligand interactions between two types of cells based on the expression levels of binding ligands in one type of cell and the expression levels of receptors in another type of cell, thereby simulating communication between cells.

First, we used immune cells, epithelial cells, and cancer cells to construct a CellChat model. We then used “CellChatDB.human” to evaluate the major signal inputs and outputs of the cell populations in all the samples. We identified overexpressed genes and subsequently identified overexpressed ligand‒receptor interactions. By summarizing the communication probabilities of all of the ligand‒receptor interactions, we computed the communication probability at the signaling pathway level and calculated the aggregated cell‒cell communication network. Although interactions were computed among all of the identified subclusters, our interpretation and analysis focused on interactions between malignant tumor cells and other cell types, including both immune and nonimmune cell types. The communication networks were ultimately depicted by using a circle plot, and signaling pathways were represented by using a bubble plot.

## Results

### Single-cell transcriptome profiling of NSCLC cells

**Fig. 2:**
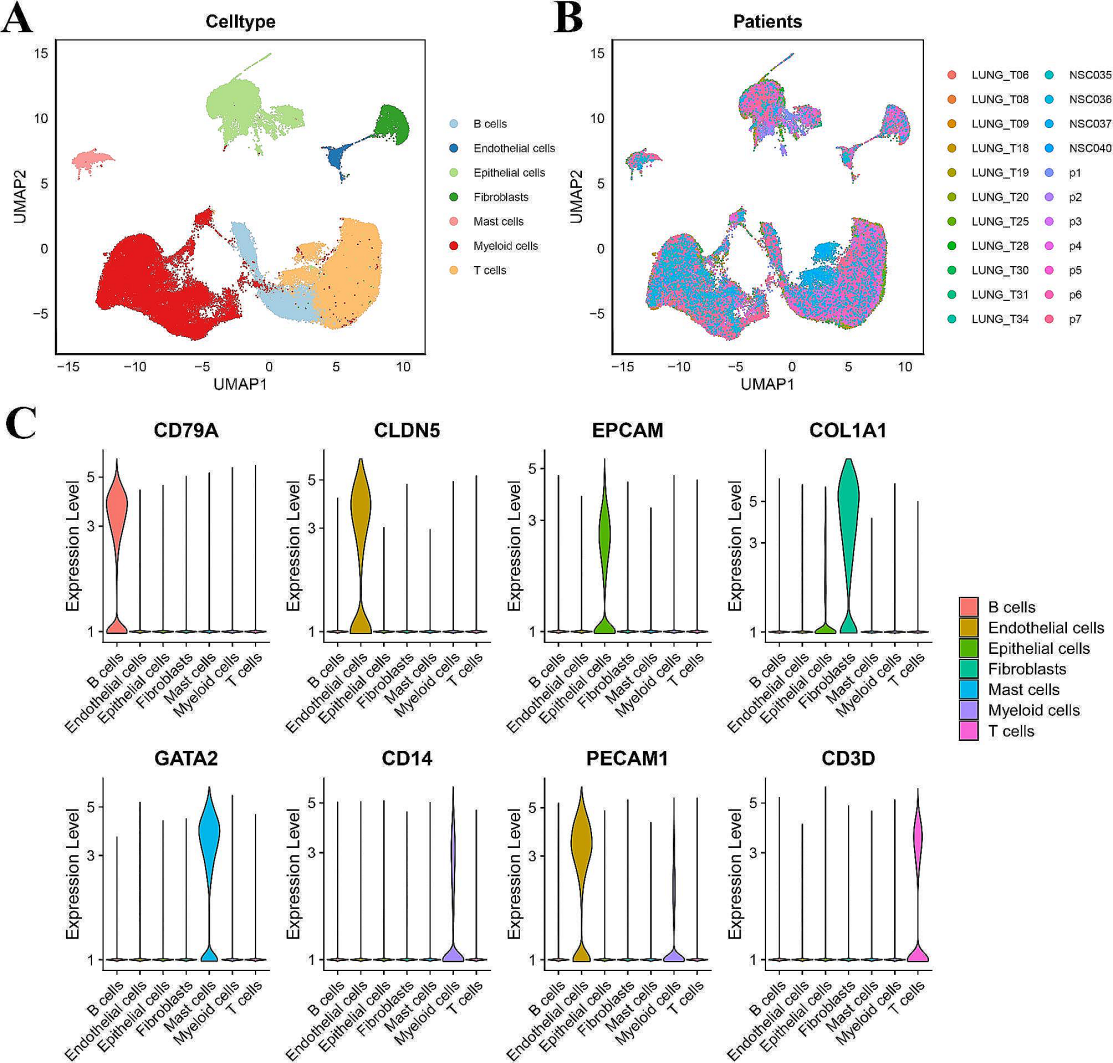
Identification of cell subsets **(A)** UMAP plot of all cells from 22 patients, colored by major cell types. **(B)** UMAP plot of all cells from 22 patients, colored by patients. **(C)** Violin plots showing the expression level of known cell-type-specific markers to demonstrate the identity of each cluster

To understand NSCLC heterogeneity at single-cell resolution and determine the changes in gene expression related to cancer progression, we collected scRNA-seq datasets from 3 studies and 22 patients. After quality filtering, 93,406 cells were retained for subsequent analysis. We identified seven major cell types, including three immune cell types (B cells [12.6%], T cells [39.6%], and myeloid cells [26.6%]) and four nonimmune cell types (endothelial cells [1.5%], epithelial cells [13.6%], mast cells [2.9%] and fibroblasts [3.2%]). Clusters were manually annotated by using normal markers and curated gene signatures that defined their identities. Specifically, T cells were distinguished by using the following marker genes: exhausted CD8 + T cells (*CD8A*, *LAG3*, and *TIGIT*), CD8 + T cells (*CD8A*, *GNLY*, *GZMA*, *GZMK*, *GZMB*, and *GZMH*), CD4 + T cells (*CCR7*, *LEF1*, *IL7R*, and *SELL*), and NK T cells (*CD8A* and *NKG7*). We initially performed graph-based cell clustering on the single-cell dataset to identify the primary cell populations of early and advanced NSCLC (Fig. 2). Principal component analysis (PCA) demonstrated that the positions of the samples at a given illness stage significantly overlapped regardless of their origin.

**Fig. 3:**
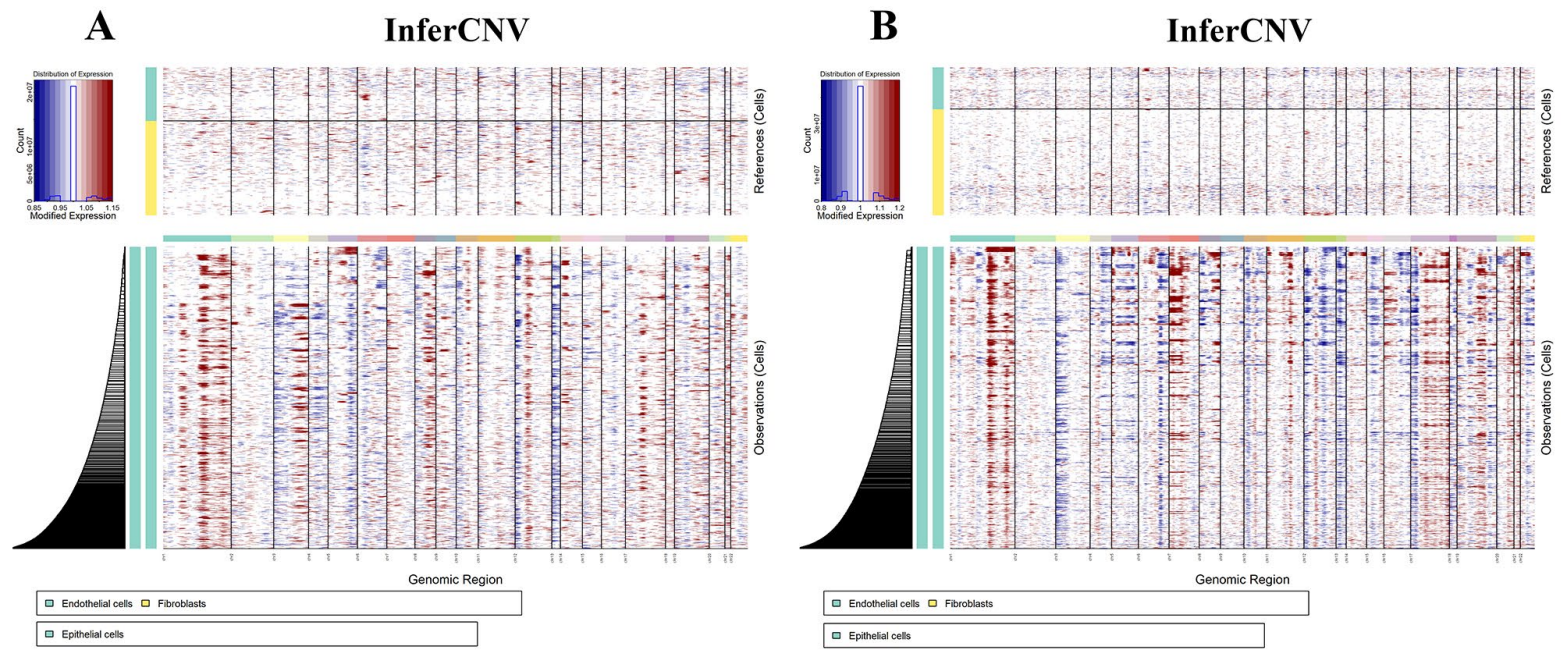
The CNV profile analysis distinguishes tumor cells. Mapping chromosome amplification (red) and deletion (blue) to each chromosome position in malignant tumor cells. **(A)** InferCNV of GSE131907 (10X sequencing platform) **(B)** InferCNV of GSE127465 and GSE136246 (inDrop sequencing platform)

In tumor tissue, epithelial cells may contain residual normal cells from the malignant tumor cell population. To define malignant cells, we calculated large-scale chromosomal CNVs in each cell type based on average expression patterns across intervals of the genome. Compared with early-stage patients, advanced-stage patients have a greater proportion of malignant tumor cells. When comparing the transcriptomes of malignant cells in early NSCLC tumors and advanced NSCLC tumors, we noticed that a series of genes were specifically expressed in malignant cells of advanced NSCLC but not in early NSCLC. The InferCNV results for the identified malignant NSCLC cells are shown in Fig. 3. GO enrichment analysis demonstrated that these genes were enriched in ATP synthesis coupled with electron transport. This result indicates that malignant tumor cells grow and proliferate rapidly and require a large amount of energy to maintain this active state. As the main energy molecule within cells, ATP plays a crucial role in supporting the growth and division of malignant tumor cells. Furthermore, large-scale chromosome CNVs were detected in advanced NSCLC tumor cells. We observed that many CNVs had already occurred in malignant cells, most notably on chromosome 6 with a deletion, which has been well described in aggressive NSCLC. In addition, chromosomes 1, 7, and 8 exhibited significant gene amplification. The consistency of these findings is reinforced by similar results reported in previous studies [37–39]. However, chromosomes 2 and 4 showed no significant CNVs. Specifically, throughout the process of cancer progression, the number of chromosome gain/loss events gradually increased in advanced NSCLC tumor cells, whereas somatic cell CNVs did not occur in other epithelial cell subtypes.

### Transcriptional trajectory of cancer cells

**Fig. 4:**
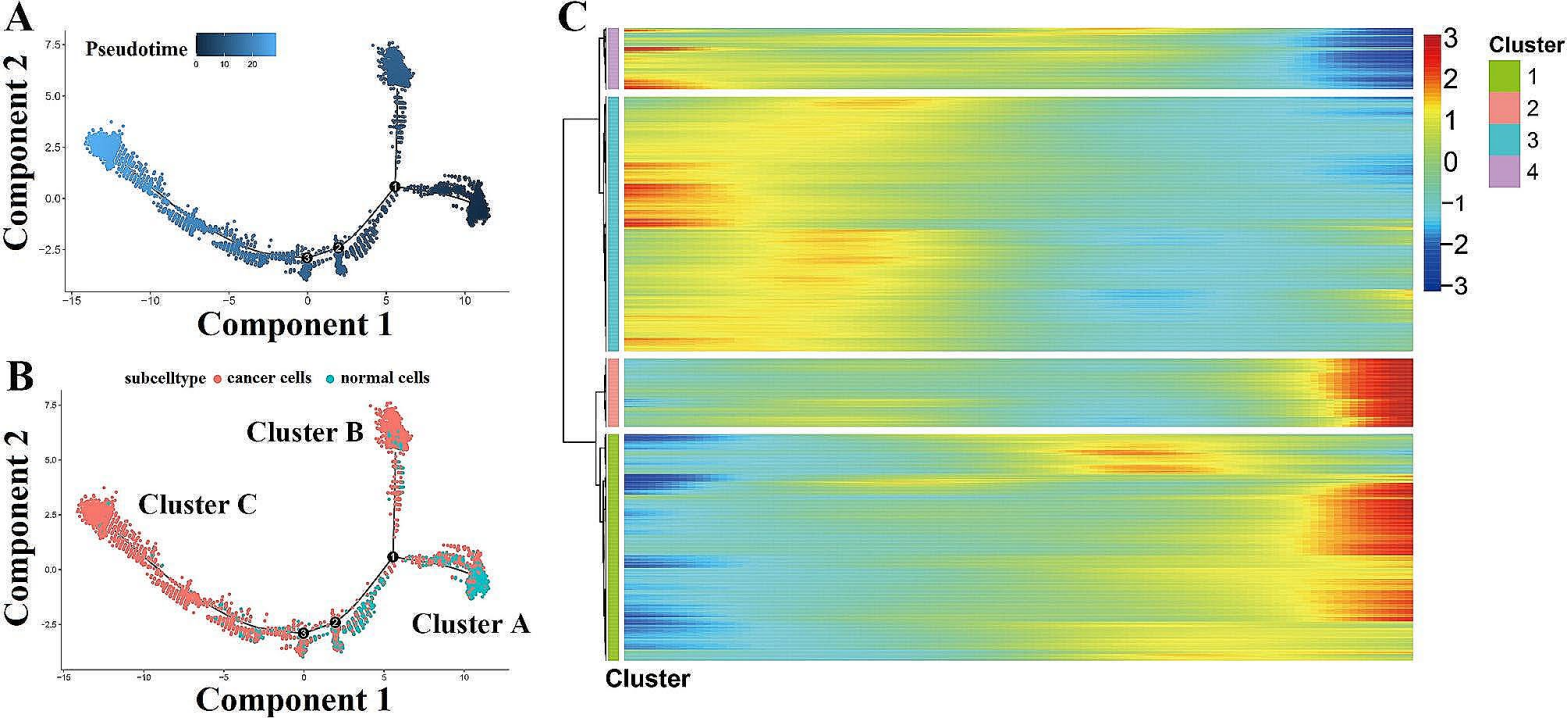
Pseudotime trajectory inferred by Monocle2 **(A)** Simulation of the development trajectory of malignant cells, colored by development stage. **(B)** Simulation of the development trajectory of malignant cells, colored by cell types. **(C)** Heatmap showing expression of representative identified genes across single cells. The color key from blue to red indicates relative expression levels from low to high

Pseudotime analysis of the epithelial cells using Monocle 2 suggested two diverging cell fates, starting at Cluster A and progressing toward Cluster B at one end and Cluster C at the other end. Cluster A mainly comprised normal cells, whereas Clusters B and C were primarily malignant epithelial cells namely cancer cells. From the perspective of time, Cluster A was found at the starting point of the pseudotime sequence, whereas Clusters B and C were found at the endpoint of the pseudotime sequence (Fig. 4A and B).

Indeed, differential gene expression analysis attributed malignant epithelial cells to the four subtypes concordant with pseudotime states. We further analyzed the gene expression patterns of all of the genes along the trajectory of malignant cell progression and identified 2037 genes with dynamic expression changes. The DEGs along the pseudotime trajectory were clustered hierarchically into four profiles (Fig. 4C).

### Functional enrichment analyses

**Fig. 5:**
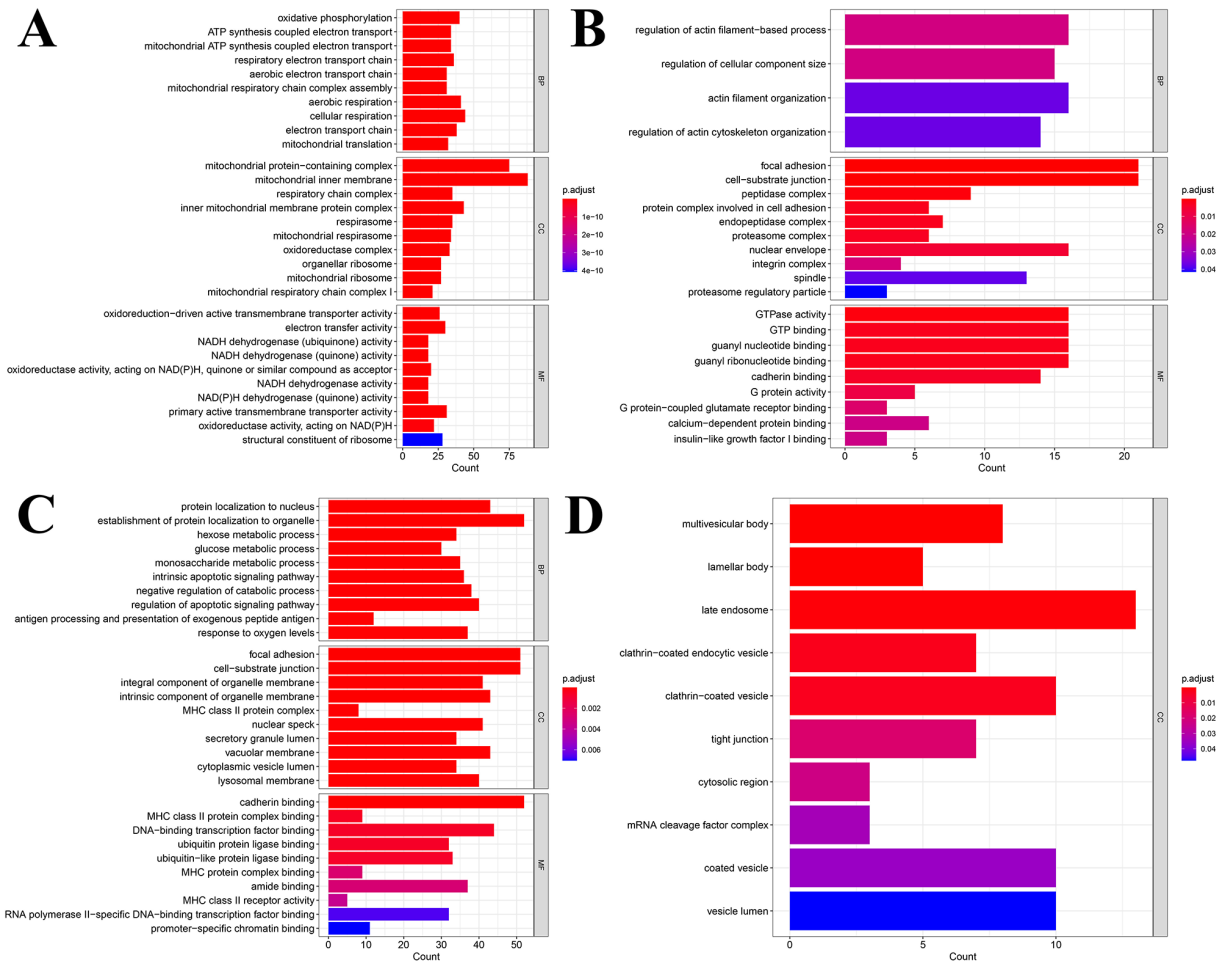
Histogram of GO enrichment analysis for differential genes across single cells. **(A)** The enrichment pathway of Cluster (1) **(B)** The enrichment pathway of Cluster (2) **(C)** The enrichment pathway of Cluster (3) **(D)** The enrichment pathway of Cluster 4

Further enrichment analyses were performed by using GO terms to compare the molecular functions and signaling pathways between the early NSCLC and advanced NSCLC groups. With pseudotime sequencing, genes (Cluster 1) with expression levels that gradually increased mainly participated in ATP synthesis coupled with electron transport (Fig. 5), whereas with pseudotime sequencing, genes (Cluster 3) with expression levels that gradually decreased participated in glycometabolism, including hexose metabolic processes, glucose metabolic processes, and monosaccharide metabolic processes. These metabolic processes contribute to the adaptation of NSCLC cells to the tumor microenvironment and facilitate immune evasion, thus promoting malignancy and resistance to therapy. We identified multiple pathways related to intracellular transport, signal transduction, and intercellular communication (including pathways related to multivesicular bodies and lamellar bodies) in Cluster 4. Dysregulation of these pathways may promote tumor growth, invasion, metastasis, and resistance to therapy [40].

### Cell–cell communication network among different cell types in early and advanced NSCLC patients

**Fig. 6:**
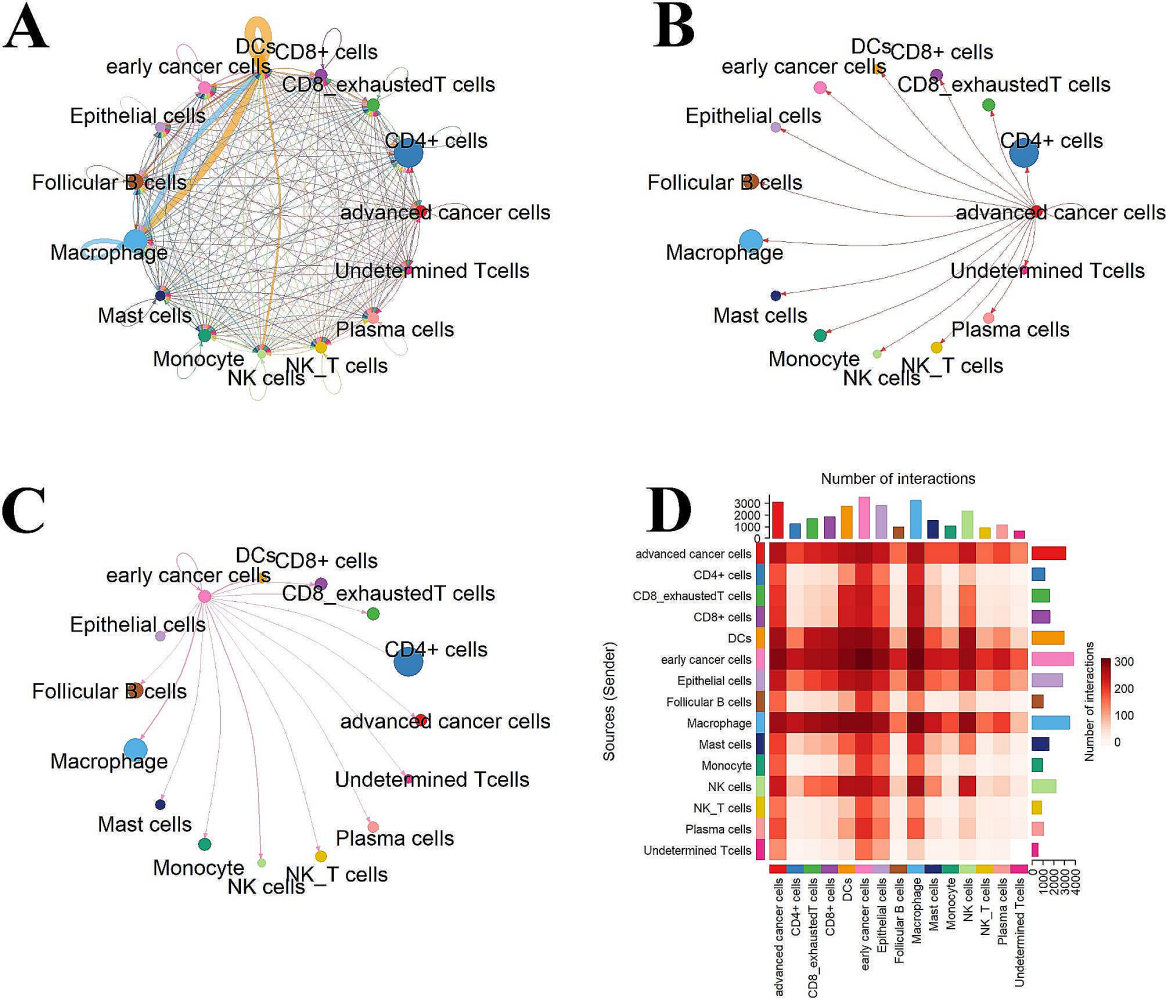
Interaction plot of tumor cells and intercellular communication networks. **(A)** The circle plot shows the inferred intercellular communication network for all cell types. **(B)** The circle plot shows the communication network between advanced NSCLC cells and other cells. **(C)** The circle plot shows the communication network between early NSCLC cells and other cells. **(D)** The heat map of the communication intensity between various cells

The cell‒cell communication network among different cell types in early and advanced NSCLC cells is shown in Fig. 6. The results showed that there were varying degrees of interactions between all types of cells. Detailed analysis of early NSCLC single-cell sequencing data demonstrated that early NSCLC cells mainly interacted with cell types, including macrophages and dendritic cells (DCs). Other types of immune cells, such as CD4+, CD8+, and exhausted CD8 + T cells, also interacted with cancer cells; however, these interactions are not as strong as interactions between DCs and macrophages with cancer cells. Tumor cells express tumor associated-antigens, which can be captured by DCs and presented to T cells [41, 42]. However, the role of macrophages in cancer is complex and diverse [43, 44]. They can fight against tumors and promote tumor growth and metastasis, depending on the characteristics of the tumor microenvironment and the functional status of the macrophages. The role of these two types of immune cells in NSCLC has also been explored [45]. As shown in Fig. 6D, advanced NSCLC cells also interacted with these immune cells; however, their interactions were less intense than those between early NSCLC cells and immune cells. We infer that this may result from immune escape in the cancer cells of the selected advanced patients. Cellular communication between advanced cancer cells and immune cells is weaker than that between early cancer cells and immune cells, which can be attributed to various factors, such as immune escape mechanisms, interference from cytokines and chemical mediators, and changes in the tumor microenvironment [13, 46, 47]. In summary, from our results on cell‒cell communication, during the development of NSCLC, tumor cells gradually develop a series of mechanisms to evade the attack of the immune system.

**Fig. 7:**
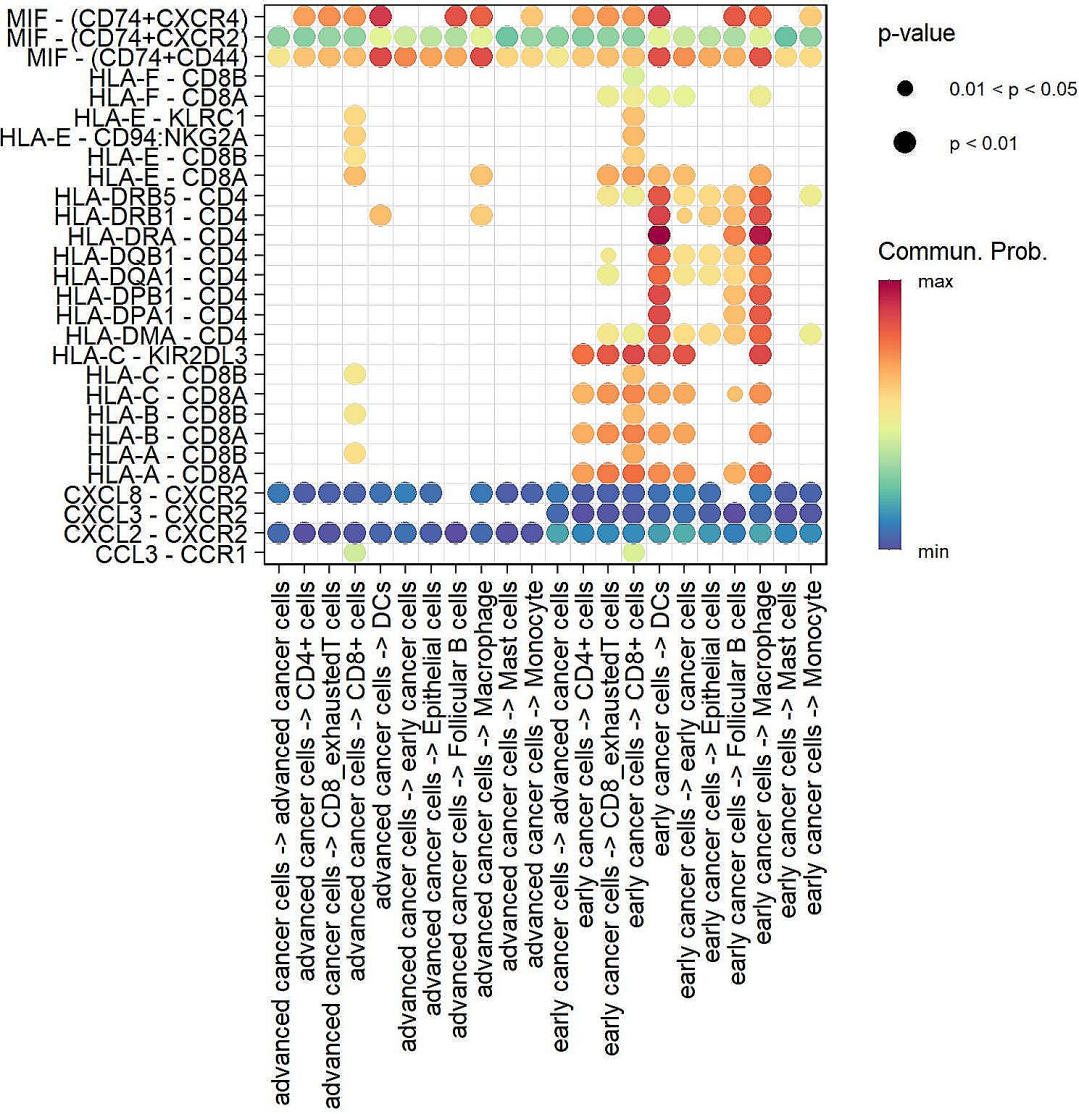
Bubble diagram showing the top receptor-ligand pairs in early and advanced NSCLC cells

Figure 7 shows the relationship between the primary receptors and ligands in the interaction between NSCLC cells and other types of cells. We found that compared to early NSCLC cells, advanced NSCLC cells interact significantly differently with different types of cells. In the early stage, DCs and macrophages inhibit the growth of malignant tumor cells. For example, early NSCLC cells strongly communicate with DCs and macrophages, which is mainly due to the interaction between *CD4* and *HLA-DRA*. However, this interaction between advanced NSCLC cells and DCs, as well as between advanced NSCLC cells and macrophages, disappears. In addition, although the cellular communication between exhausted CD8 + T cells and NSCLC cells is not strong, the interaction between NSCLC cells and exhausted CD8 + T cells changes to a lesser degree in both the early and advanced stages of NSCLC. The interaction between NSCLC cells and exhausted CD8 + T cells is mainly dependent on the interaction between CD8 + T cells (*CD8A* and *CD8B*) and *HLA-A*, *HLA-B*, and *HLA-C*.

### Gene expression programs obtained using cNMF

**Fig. 8:**
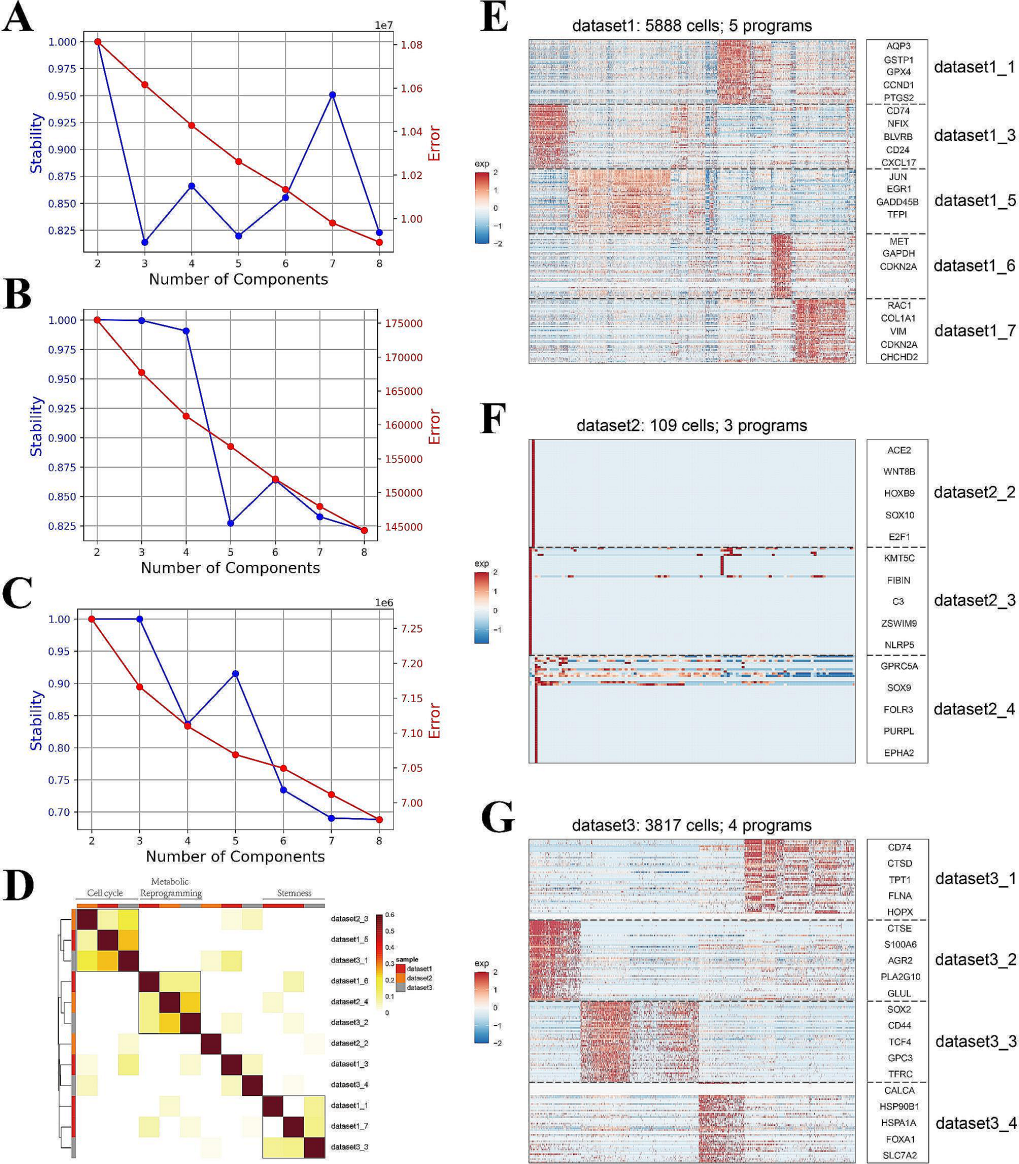
(A) K-selection plot of dataset1. **(B)** K-selection plot of dataset2. **(C)** K-selection plot of dataset3. **(D)** Pearson correlation matrix for selected programs. **(E)** Heatmap showing correlation of programs derived from cNMF analysis of single cell dataset1 **(F)** Heatmap showing correlation of programs derived from cNMF analysis of single cell dataset2. **(G)** Heatmap showing correlation of programs derived from cNMF analysis of single cell dataset3

We performed cNMF analysis on cancer cells from two datasets dataset1 (GSE131907), dataset2 (GSE136246), and dataset3 (GSE127465). We used K-selection plots to determine the most suitable value for K (Fig. 8A, B and C). Finally, we obtained a total of 12 expression programs. According to the heatmap (Fig. 8E, F and G), there were significant differences in the activity of the cells in these different expression programs. The annotated genes have a greater weight in the corresponding expression programs, which indicates that these genes may play a crucial role in defining these expression programs.

To explore the interconnections among expression programs, Pearson correlation analysis was employed to group highly correlated expression programs into distinct modules (Fig. 8D). This process yielded a total of three modules. By conducting enrichment analysis on the top 50 genes with the highest weight in each expression program, relevant pathways were identified. These pathways were used to define three modules: the metabolic reprogramming module, cell cycle module, and cell stemness module. The top 50 genes in each program and the pathways enriched by these genes are listed in Supplementary Table S2.

### Evaluating the importance of genes with pseudotime clustering using XGBoost

When comparing the transcriptomes of malignant cells in early NSCLC tumors and advanced NSCLC tumors, we noticed that a series of genes were specifically expressed in malignant cells of advanced NSCLC but not in early NSCLC. The results of the pseudotime analysis also indicated that the expression levels of some genes change with the development of the disease. Therefore, we used XGBoost to build candidate gene models with the training set and evaluated them on the validation set. The accuracy of the model reached 0.973. The model utilizes the importance of XGBoost as a foundation for gene screening. Out of 2037 genes, 596 ultimately yielded importance scores. These identified genes can be further explored to elucidate the characteristics influencing the prediction model. Using cNMF analysis, we identified a total of 731 specific genes. Subsequently, we compared this gene set with those identified by XGBoost. The overlap between the gene sets obtained through both methods demonstrated a total of 136 genes (Supplementary Table S3). *HN1*, *AQP3*, and *GSTP1* were the top three genes in the rankings.

### Proteins overexpressed in NSCLC

**Fig. 9:**
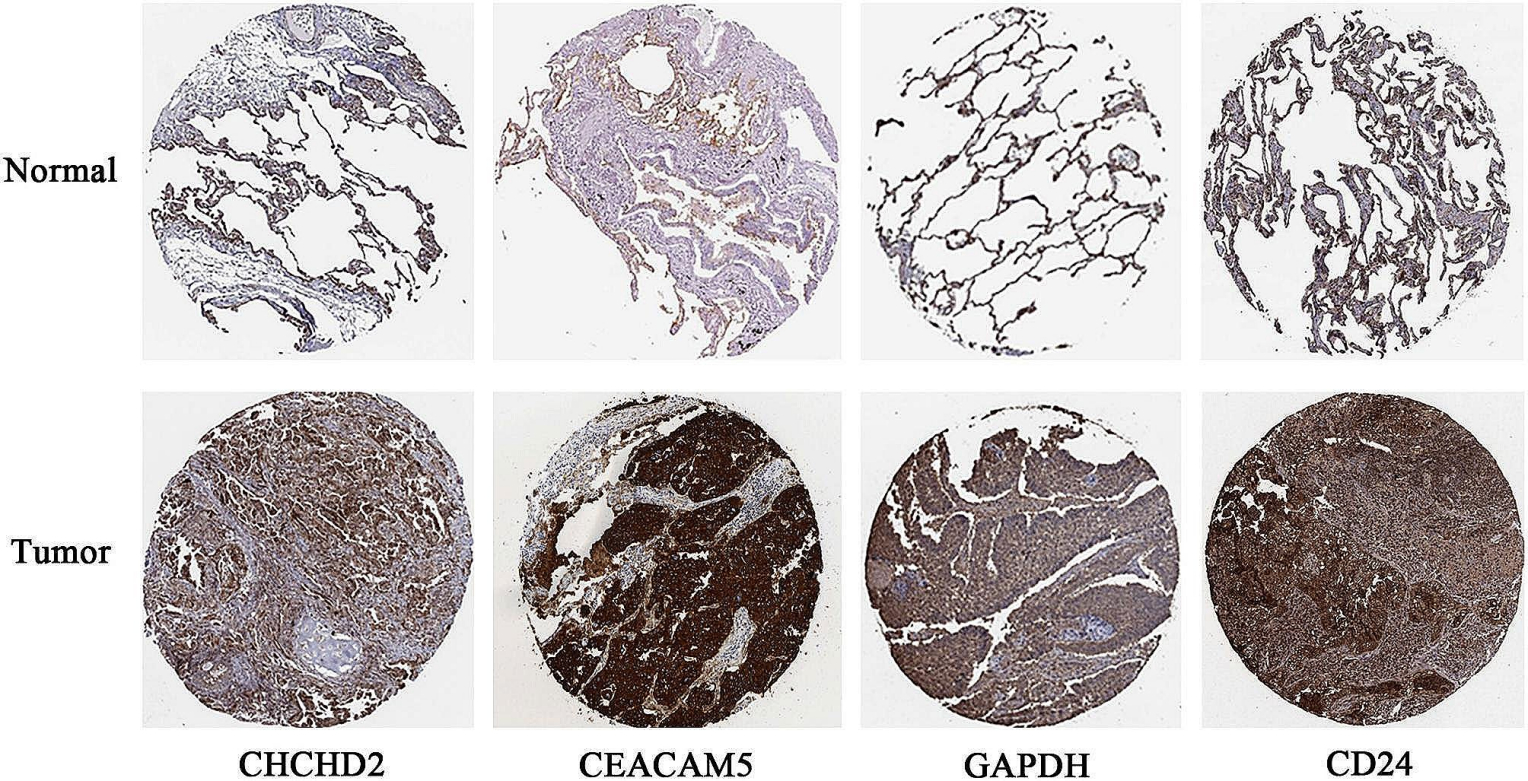
Immunohistochemical staining analysis of *CHCHD2*, *CEACAM5*, *GAPDH*, and *CD24* in normal lung and lung cancer tissues

We searched for the screened genes in the HPA database. In immunohistochemistry experiments, the expression levels of multiple genes, including *CHCHD2*, *CEACAM5*, *GAPDH*, and *CD24*, were significantly upregulated in lung cancer tissues compared to normal tissues (Fig. 9).

### Construction of the prognostic risk score

**Fig. 10:**
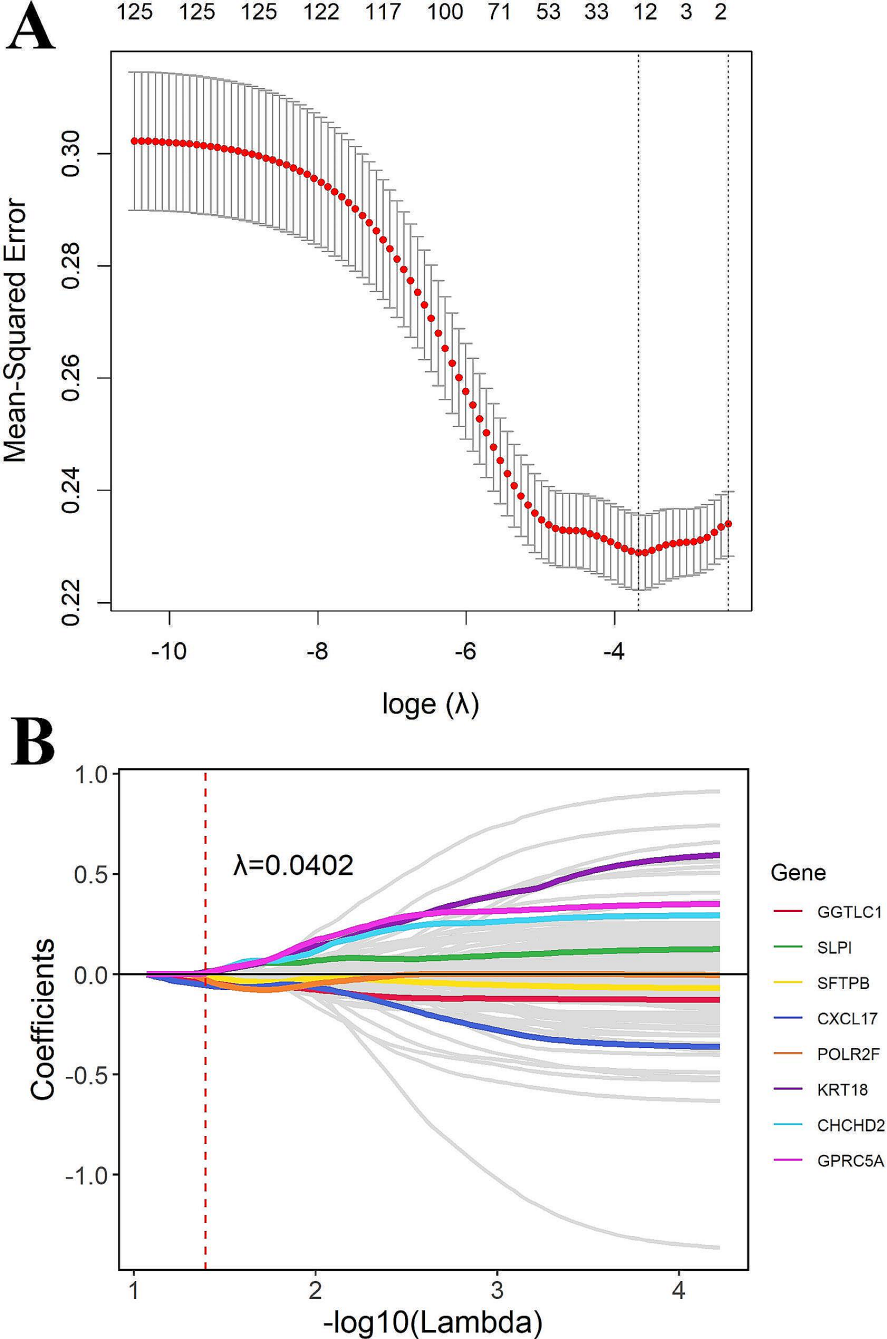
Identification of prognostic biomarkers related to the temporal heterogeneity of NSCLC. **(A)** Determination of the number of factors by the LASSO algorithm. **(B)** The genes obtained from LASSO regression downscaling

**Fig. 11:**
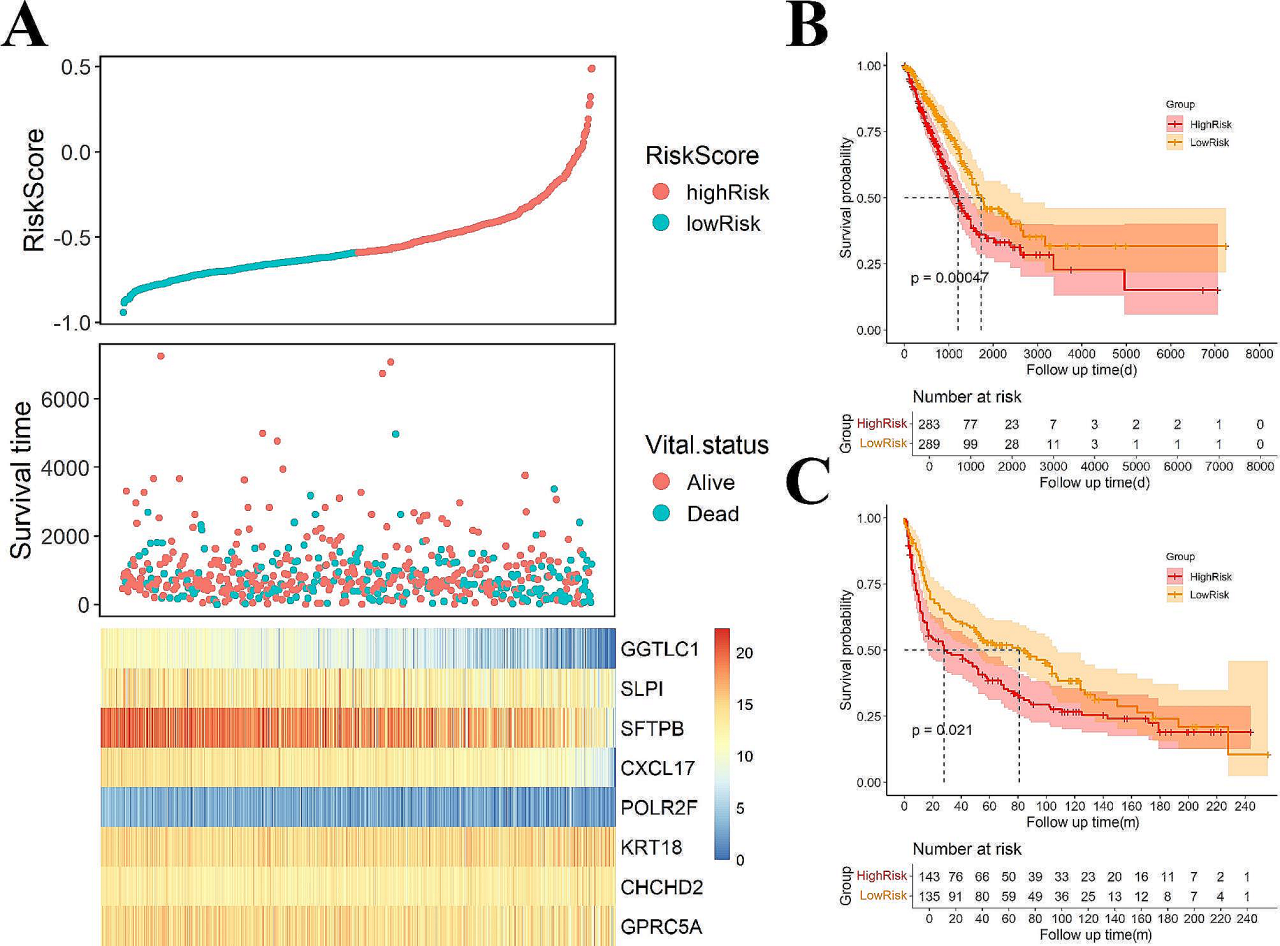
**(A)** The distribution of risk score and survival status and the heatmap of 8 genes in the TCGA_LUAD cohort. **(B)** Kaplan–Meier curve depicts the OS difference between highrisk and lowrisk groups in TCGA. **(C)** Kaplan–Meier curve depicts the OS difference between highrisk and lowrisk groups in GSE30219.

To promote the clinical application of identifying genes in evaluating survival prognosis, we used LASSO feature selection to further select the most important feature from all of the genes (Fig. 10). The optimal model included *GGTLC1*, *SLPI*, *SFTPB*, *CXCL17*, *POLR2F*, *KRT18*, *CHCHD2*, and *GPRC5A*. The coefficients of each gene are listed in Supplementary Table S4. Furthermore, the prognostic value of these genes was also evaluated. To further assess the robustness of the risk score, we selected an independent dataset (GSE30219) to validate the prognostic predictive power of the risk score. In both datasets (TCGA and GSE30219), the KM curve showed that the high-risk group had significantly worse overall survival (Fig. 11).

## Discussion

NSCLC is a highly heterogeneous cancer with high mortality and recurrence rates. In this study, we integrated the scRNA-seq transcriptional profiles of NSCLC at various disease stages to discover novel features of this disease. Single-cell sequencing data have demonstrated the developmental trajectory leading from early NSCLC to advanced NSCLC. The results will assist us in identifying the essential genes and signaling pathways that influence the development of this illness. Additional analysis based on CNV suggested that in comparison to early NSCLC, advanced NSCLC exhibits a heightened degree of epithelial cell degeneration.

Through pseudotime analysis, we identified a correlation between 2037 genes and the development of malignant epithelial cells in NSCLC. These genes are classified into four clusters. Using gene enrichment analysis, we found that ATP synthesis, metabolic processes, and cellular transport are key driving factors for the malignant progression of NSCLC. Pseudotime analysis has also demonstrated many genes related to the dynamic changes in NSCLC, and some genes are expected to be used to indicate the developmental status of NSCLC. We evaluated the importance of these genes by using the machine learning algorithm XGBoost. Using cNMF, we categorized NSCLC single cells into three distinct modules, including the metabolic reprogramming module, cell cycle module, and cell stemness module. This further confirms that metabolic issues are worthy of attention in the development of NSCLC, which is consistent with previous research [48–50].

The metabolic reprogramming module, which is characterized by the enrichment of pathways such as glycolysis, heme metabolism, and cholesterol homeostasis, plays a crucial role in fueling the rapid growth and proliferation of cancer cells. Dysregulation of these metabolic pathways is a hallmark of cancer, including NSCLC, wherein altered metabolism contributes to tumor progression, metastasis, and therapy resistance [51–53]. Similarly, the cell cycle module, which was enriched with genes involved in cell cycle regulation, reflects the dysregulated proliferation characteristic of NSCLC. Aberrant cell cycle progression is a hallmark of cancer, and the dysregulated expression of cell cycle genes promotes uncontrolled cell proliferation and tumor growth [54, 55]. Additionally, the enrichment of genes associated with stem cell-like properties (including epithelial–mesenchymal transition and estrogen response) in the stemness module highlights the presence of cancer stem cells (CSCs) in NSCLC. CSCs are a subpopulation of tumor cells with enhanced tumorigenicity and therapeutic resistance that contribute to tumor metastasis and recurrence [56, 57].

Subsequently, we identified and extracted significant characteristic genes associated with these subtypes. *MET*, *GAPDH*, and *GLUL* were identified as being highly variable in the cNMF analysis, and they have been confirmed to participate in metabolic processes. *MET* influences cellular metabolism by activating signaling pathways that regulate glucose uptake and utilization. *GAPDH* is a key enzyme in glycolysis. *GLUL* encodes glutamine synthetase, which supports cancer cell proliferation by promoting glutamine metabolism. Together, these genes contribute to tumor growth, survival, and metastasis through their roles in cellular metabolism and signaling pathways. Furthermore, previous studies have also established their correlation with NSCLC [58–60]. The expression of *EGR1* and *JUN* influences cancer progression by regulating cell cycle processes and promoting cell proliferation. Both *EGR1* and *JUN* are implicated in NSCLC, thus driving oncogenic transformation and disease [61, 62].

Although cNMF identified many highly variable genes, we conducted a comparison with the results from XGBoost. This approach aids us in the more effective identification of key biomarkers. The XGBoost evaluation yielded 596 genes, and the intersection with genes obtained from cNMF analysis resulted in a set of 136 genes. The top three genes in the rankings were *HN1*, *AQP3*, and *GSTP1*. Previous research has established a significant association between *AQP3* and *GSTP1* and the onset, progression, and therapeutic resistance of NSCLC [63–66]. These molecules exhibit promise as being prospective biomarkers or therapeutic targets. Conversely, investigations into the interplay between *HN1* and NSCLC are limited. Our novel findings indicate the potential involvement of *HN1* in the genesis and progression of NSCLC, thus warranting further exploration into its mechanistic contributions to disease pathology.

We also investigated the disparities in the tumor microenvironment between early-stage and advanced-stage NSCLC patients. By examining individual cells within the tumor microenvironment, we were able to identify subtle differences in gene expression profiles that may have been overlooked in previous bulk tissue analyses.

Cell‒cell interaction analysis demonstrated interactions between immune cells (DCs and macrophages) and cancer cells in both early and advanced NSCLC. However, the interaction between immune cells and early NSCLC tumor cells was stronger than that between immune cells and advanced NSCLC tumor cells. In the early stages of NSCLC, the number of tumor cells is relatively small, thus making it easier for DCs and macrophages to detect and recognize these abnormal cells. As tumors develop, the number of cancer cells increases; however, due to the immune escape mechanism, the ability of immune cells to monitor cancer cells may decrease [67–69]. This factor may weaken the immune response of DCs and macrophages to advanced cancer cells. We observed no interaction between HLA-DRA and CD4, both between tumor cells and macrophages and between tumor cells and DCs, which may explain this phenomenon in advanced NSCLC patients. Furthermore, as tumors develop, the heterogeneity of tumor cells increases, and some subgroups may become more resistant to immunotherapy. These subgroups may dominate in the advanced stage, thus limiting the role of immune cells. In summary, intercellular interactions indicate a close relationship between immune cell dynamics and the molecular characteristics of cancer cells, which may determine the prognosis and treatment response of NSCLC patients.

We established a Lasso model by using the identified genes associated with temporal heterogeneity in NSCLC. The optimal model included genes such as *GGTLC1*, *SLPI*, *SFTPB*, *CXCL17*, *POLR2F*, *KRT18*, *CHCHD2*, and *GPRC5A*. Immunohistochemical data demonstrated elevated expression levels of *CHCHD2* in lung cancer tissues compared to normal tissues [70, 71]. However, the negative Lasso coefficient of *CXCL17* suggested that its low expression may increase disease risk. These results demonstrate the efficacy of our feature selection approach in identifying key genes associated with NSCLC progression.

Admittedly, limitations existed in this research. We employed various methods to identify marker genes and pathways associated with temporal heterogeneity in NSCLC at the single-cell level. Despite an adequate number of single cells, the sparsity inherent in single-cell sequencing data remains a concern that could impact the accuracy of machine learning prediction results. Additionally, although our study identified key gene signatures associated with NSCLC progression, we did not perform functional validation of these genes. Experimental validation, such as in vitro and in vivo assays, is necessary to elucidate the biological roles of these genes and validate their potential as therapeutic targets or prognostic markers. Future research should aim to validate our findings in independent cohorts and explore the functional significance of the identified genes in NSCLC progression.

## Conclusion

In conclusion, our study provides valuable insights into the temporal heterogeneity of NSCLC and highlights significant genomic disparities between its early and advanced stages. Through comprehensive single-cell transcriptomic analysis, we identified distinct cancer cell subtypes and delineated temporal gene expression patterns associated with NSCLC progression. The establishment of a risk score model based on these genes offers potential clinical utility in cancer risk assessment and prognostication for NSCLC patients. Our findings underscore the importance of understanding the molecular mechanisms underlying NSCLC progression and provide a foundation for further research aimed at improving clinical management and personalized treatment strategies for NSCLC patients. Furthermore, our approach has the potential to provide valuable insights into the temporal dynamics of biological systems and can be used to identify key biological pathways.

### Electronic supplementary material

Below is the link to the electronic supplementary material.

Supplementary Material 1

Supplementary Material 2

Supplementary Material 3

Supplementary Material 4

Supplementary Material 5

## Data Availability

No datasets were generated or analysed during the current study.

## Declarations

### Note

Ethical Statement: The authors are accountable for all aspects of the work in ensuring that questions related to the accuracy or integrity of any part of the work are appropriately investigated and resolved.

## References

[1] Zhang H, Jiang H, Zhu L, Li J, Ma S (2021). Cancer-associated fibroblasts in non-small cell lung cancer: recent advances and future perspectives. Cancer Lett.

[2] Rotow J, Bivona TG (2017). Understanding and targeting resistance mechanisms in NSCLC. Nat Rev Cancer.

[3] Jamal-Hanjani M, Wilson GA, McGranahan N, Birkbak NJ, Watkins TBK, Veeriah S, Shafi S, Johnson DH, Mitter R, Rosenthal R (2017). Tracking the evolution of Non-small-cell Lung Cancer. N Engl J Med.

[4] de Bruin EC, McGranahan N, Mitter R, Salm M, Wedge DC, Yates L, Jamal-Hanjani M, Shafi S, Murugaesu N, Rowan AJ (2014). Spatial and temporal diversity in genomic instability processes defines lung cancer evolution. Science.

[5] Frankell AM, Dietzen M, Al Bakir M, Lim EL, Karasaki T, Ward S, Veeriah S, Colliver E, Huebner A, Bunkum A (2023). The evolution of lung cancer and impact of subclonal selection in TRACERx. Nature.

[6] Martinez-Ruiz C, Black JRM, Puttick C, Hill MS, Demeulemeester J, Larose Cadieux E, Thol K, Jones TP, Veeriah S, Naceur-Lombardelli C (2023). Genomic-transcriptomic evolution in lung cancer and metastasis. Nature.

[7] Qiao M, Jiang T, Liu X, Mao S, Zhou F, Li X, Zhao C, Chen X, Su C, Ren S, Zhou C (2021). Immune checkpoint inhibitors in EGFR-Mutated NSCLC: Dusk or Dawn?. J Thorac Oncol.

[8] Harrison PT, Vyse S, Huang PH (2020). Rare epidermal growth factor receptor (EGFR) mutations in non-small cell lung cancer. Semin Cancer Biol.

[9] Ferrer I, Zugazagoitia J, Herbertz S, John W, Paz-Ares L, Schmid-Bindert G (2018). KRAS-Mutant non-small cell lung cancer: from biology to therapy. Lung Cancer.

[10] Chabon JJ, Simmons AD, Lovejoy AF, Esfahani MS, Newman AM, Haringsma HJ, Kurtz DM, Stehr H, Scherer F, Karlovich CA (2016). Circulating tumour DNA profiling reveals heterogeneity of EGFR inhibitor resistance mechanisms in lung cancer patients. Nat Commun.

[11] Dearden S, Stevens J, Wu YL, Blowers D (2013). Mutation incidence and coincidence in non small-cell lung cancer: meta-analyses by ethnicity and histology (mutMap). Ann Oncol.

[12] Cords L, Engler S, Haberecker M, Rüschoff JH, Moch H, de Souza N, Bodenmiller B. Cancer-associated fibroblast phenotypes are associated with patient outcome in non-small cell lung cancer. 10.1016/j.ccell.2023.12.021 PMC10929690 38242124

[13] Wu F, Fan J, He Y, Xiong A, Yu J, Li Y, Zhang Y, Zhao W, Zhou F, Li W (2021). Single-cell profiling of tumor heterogeneity and the microenvironment in advanced non-small cell lung cancer. Nat Commun.

[14] Dong N, Shi L, Wang DC, Chen C, Wang X. Role of epigenetics in lung cancer heterogeneity and clinical implication. 10.1016/j.semcdb.2016.08.029 27575638

[15] Hua X, Zhao W, Pesatori AC, Consonni D, Caporaso NE, Zhang T, Zhu B, Wang M, Jones K, Hicks B (2020). Genetic and epigenetic intratumor heterogeneity impacts prognosis of lung adenocarcinoma. Nat Commun.

[16] Lei Y, Tang R, Xu J, Wang W, Zhang B, Liu J, Yu X, Shi S (2021). Applications of single-cell sequencing in cancer research: progress and perspectives. J Hematol Oncol.

[17] Rozenblatt-Rosen O, Regev A, Oberdoerffer P, Nawy T, Hupalowska A, Rood JE, Ashenberg O, Cerami E, Coffey RJ, Demir E (2020). The human tumor Atlas Network: charting Tumor transitions across Space and Time at single-cell resolution. Cell.

[18] Wu Y, Yang S, Ma J, Chen Z, Song G, Rao D, Cheng Y, Huang S, Liu Y, Jiang S (2022). Spatiotemporal Immune Landscape of Colorectal Cancer Liver Metastasis at single-cell level. Cancer Discov.

[19] Hao Y, Hao S, Andersen-Nissen E, Mauck WM, Zheng S, Butler A, Lee MJ, Wilk AJ, Darby C, Zager M (2021). Integrated analysis of multimodal single-cell data. Cell.

[20] Patel AP, Tirosh I, Trombetta JJ, Shalek AK, Gillespie SM, Wakimoto H, Cahill DP, Nahed BV, Curry WT, Martuza RL et al. Single-cell RNA-seq highlights intratumoral heterogeneity in primary glioblastoma. 10.1126/science.1254257 PMC4123637 24925914

[21] Fan JA-O, Lee HO, Lee S, Ryu DE, Lee S, Xue C, Kim SJ, Kim K, Barkas N, Park PJ et al. Linking transcriptional and genetic tumor heterogeneity through allele analysis of single-cell RNA-seq data. 10.1101/gr.228080.117 PMC6071640 29898899

[22] Trapnell C, Cacchiarelli D, Grimsby J, Pokharel P, Li S, Morse M, Lennon NJ, Livak KJ, Mikkelsen TS, Rinn JL (2014). The dynamics and regulators of cell fate decisions are revealed by pseudotemporal ordering of single cells. Nat Biotechnol.

[23] Cao J, Spielmann M, Qiu X, Huang X, Ibrahim DM, Hill AJ, Zhang F, Mundlos S, Christiansen L, Steemers FJ (2019). The single-cell transcriptional landscape of mammalian organogenesis. Nature.

[24] Croizer H, Mhaidly R, Kieffer YA-O, Gentric GA-O, Djerroudi L, Leclere RA-O, Pelon F, Robley C, Bohec MA-OX, Meng A et al. Deciphering the spatial landscape and plasticity of immunosuppressive fibroblasts in breast cancer. 10.1038/s41467-024-47068-z PMC10984943 38561380

[25] Wang M, Liu Y, Sun R, Liu F, Li J, Yan L, Zhang J, Xie X, Li D, Wang Y (2024). Single-nucleus multi-omic profiling of human placental syncytiotrophoblasts identifies cellular trajectories during pregnancy. Nat Genet.

[26] Desai TJ, Brownfield DG, Krasnow MA (2014). Alveolar progenitor and stem cells in lung development, renewal and cancer. Nature.

[27] Kotliar D, Veres A, Nagy MA, Tabrizi S, Hodis E, Melton DA, Sabeti PC. Identifying gene expression programs of cell-type identity and cellular activity with single-cell RNA-Seq. Elife 2019; 8. 10.7554/eLife.43803 PMC6639075 31282856

[28] Ni Y, Lu M, Li M, Hu X, Li F, Wang Y, Xue D. Unraveling the underlying pathogenic factors driving nonalcoholic steatohepatitis and hepatocellular carcinoma: an in-depth analysis of prognostically relevant gene signatures in hepatocellular carcinoma. 10.1186/s12967-024-04885-6 PMC10795264 38238845

[29] Deng X, Li M, Deng S, Wang L (2022). Hybrid gene selection approach using XGBoost and multi-objective genetic algorithm for cancer classification. Med Biol Eng Comput.

[30] Li H, Shi L, Gao W, Zhang Z, Zhang L, Zhao Y, Wang G. dPromoter-XGBoost: detecting promoters and strength by combining multiple descriptors and feature selection using XGBoost. 10.1016/j.ymeth.2022.01.001 34998983

[31] Panagiotopoulos KA-OX, Korfiati A, Theofilatos KA-O, Hurwitz PA-OX, Deriu MA-O, Mavroudi SA-O. MEvA-X: a hybrid multiobjective evolutionary tool using an XGBoost classifier for biomarkers discovery on biomedical datasets. LID – 10.1093/bioinformatics/btad384 [doi] LID - btad384. 10.1093/bioinformatics/btad384 PMC10354005 37326976

[32] Thul PJ, Lindskog CA-O. The human protein atlas: A spatial map of the human proteome. 10.1002/pro.3307 PMC5734309 28940711

[33] Jin S, Guerrero-Juarez CF, Zhang L, Chang I, Ramos R, Kuan CH, Myung P, Plikus MV, Nie Q (2021). Inference and analysis of cell-cell communication using CellChat. Nat Commun.

[34] Lake BB, Menon R, Winfree S, Hu Q, Melo Ferreira R, Kalhor K, Barwinska D, Otto EA, Ferkowicz M, Diep D (2023). An atlas of healthy and injured cell states and niches in the human kidney. Nature.

[35] Liu X, Song J, Zhang H, Liu X, Zuo F, Zhao Y, Zhao Y, Yin X, Guo X, Wu X et al. Immune checkpoint HLA-E:CD94-NKG2A mediates evasion of circulating tumor cells from NK cell surveillance. 10.1016/j.ccell.2023.01.001 36706761

[36] Reichart DA-O, Lindberg EA-OX, Maatz HA-O, Miranda AMA, Viveiros AA-O, Shvetsov NA-O, Gärtner AA-O, Nadelmann EA-O, Lee MA-O, Kanemaru KA-O et al. Pathogenic variants damage cell composition and single cell transcription in cardiomyopathies. 10.1126/science.abo1984 PMC9528698 35926050

[37] He H, Ma H, Chen Z, Chen J, Wu D, Lv X, Zhu J. Chromosomal Copy Number Variation predicts EGFR-TKI response and prognosis for patients with Non-small Cell Lung Cancer. 10.2147/PGPM.S418320 PMC10505391 37724294

[38] Voutsadakis IA-O. Characteristics and prognosis of 8p11.23-Amplified squamous lung carcinomas. LID – 10.3390/jcm12051711 [doi] LID – 1711. 10.3390/jcm12051711 PMC10002535 36902501

[39] Camidge DR, Otterson GA, Clark JW, Ignatius Ou SH, Weiss J, Ades S, Shapiro GI, Socinski MA, Murphy DA, Conte U et al. Crizotinib in Patients With MET-Amplified NSCLC. 10.1016/j.jtho.2021.02.010 33676017

[40] Liu CA-OX, Liu D, Wang SA-O, Gan L, Yang X, Ma CA-O. Identification of the SNARE complex that mediates the fusion of multivesicular bodies with the plasma membrane in exosome secretion. 10.1002/jev2.12356 PMC10497535 37700095

[41] Veglia F, Gabrilovich DI (2017). Dendritic cells in cancer: the role revisited. Curr Opin Immunol.

[42] Wculek SK, Cueto FJ, Mujal AM, Melero I, Krummel MF, Sancho D (2020). Dendritic cells in cancer immunology and immunotherapy. Nat Rev Immunol.

[43] Christofides A, Strauss L, Yeo A, Cao C, Charest A, Boussiotis VA (2022). The complex role of tumor-infiltrating macrophages. Nat Immunol.

[44] Mantovani A, Allavena P, Marchesi F, Garlanda C (2022). Macrophages as tools and targets in cancer therapy. Nat Rev Drug Discov.

[45] Carbone DP, Gandara DR, Antonia SJ, Zielinski C, Paz-Ares L (2015). Non-small-cell Lung Cancer: role of the Immune System and potential for Immunotherapy. J Thorac Oncol.

[46] Anichini A, Perotti VE, Sgambelluri F, Mortarini R. Immune escape mechanisms in non small cell Lung Cancer. Cancers (Basel) 2020; 12. 10.3390/cancers12123605 PMC7761620 33276569

[47] Ramachandran S, Verma AK, Dev K, Goyal Y, Bhatt D, Alsahli MA, Rahmani AH, Almatroudi A, Almatroodi SA, Alrumaihi F, Khan NA. Role of Cytokines and Chemokines in NSCLC Immune Navigation and Proliferation. Oxid Med Cell Longev. 2021; 2021:5563746. 10.1155/2021/5563746 PMC8313354 34336101

[48] Sun Z, Zhang R, Zhang X, Sun Y, Liu P, Francoeur N, Han L, Lam WY, Yi Z, Sebra R et al. LINE-1 promotes tumorigenicity and exacerbates tumor progression via stimulating metabolism reprogramming in non-small cell lung cancer. 10.1186/s12943-022-01618-5 PMC9288060 35842613

[49] Dowling CM, Zhang H, Chonghaile TN, Wong KK. Shining a light on metabolic vulnerabilities in non-small cell lung cancer. 10.1016/j.bbcan.2020.188462 PMC7836022 33130228

[50] Majem B, Nadal E, Muñoz-Pinedo C. Exploiting metabolic vulnerabilities of Non small cell lung carcinoma. 10.1016/j.semcdb.2019.06.004 31238096

[51] Huang Y, Chen Z, Lu T, Bi G, Li M, Liang J, Hu Z, Zheng Y, Yin J, Xi J et al. HIF-1α switches the functionality of TGF-β signaling via changing the partners of smads to drive glucose metabolic reprogramming in non-small cell lung cancer. 10.1186/s13046-021-02188-y PMC8690885 34930376

[52] Dey PA-OX, Kimmelman AC, DePinho RA-OX. Metabolic Codependencies in the Tumor Microenvironment. 10.1158/2159-8290.CD-20-1211 PMC8102306 33504580

[53] Sohoni S, Ghosh P, Wang T, Kalainayakan SA-O, Vidal C, Dey S, Konduri PC, Zhang L. Elevated Heme Synthesis and Uptake Underpin intensified oxidative metabolism and tumorigenic functions in Non-small Cell Lung Cancer cells. 10.1158/0008-5472.CAN-18-2156 30902795

[54] Matthews HK, Bertoli CA-O, de Bruin RA-O. Cell cycle control in cancer. 10.1038/s41580-021-00404-3 34508254

[55] Suski JM, Braun M, Strmiska V, Sicinski P. Targeting cell-cycle machinery in cancer. 10.1016/j.ccell.2021.03.010 PMC8206013 33891890

[56] Huang Q, Liu L, Xiao D, Huang Z, Wang W, Zhai K, Fang X, Kim J, Liu J, Liang W et al. CD44(+) lung cancer stem cell-derived pericyte-like cells cause brain metastases through GPR124-enhanced trans-endothelial migration. 10.1016/j.ccell.2023.07.012 37595587

[57] Yang L, Shi P, Zhao G, Xu J, Peng W, Zhang J, Zhang G, Wang X, Dong Z, Chen F, Cui H. Targeting cancer stem cell pathways for cancer therapy. 10.1038/s41392-020-0110-5 PMC7005297 32296030

[58] Remon J, Hendriks LEL, Mountzios G, García-Campelo R, Saw SPL, Uprety D, Recondo G, Villacampa G, Reck M. MET alterations in NSCLC-Current Perspectives and Future Challenges. 10.1016/j.jtho.2022.10.015 36441095

[59] Ouyang XA-O, Zhu R, Lin L, Wang X, Zhuang Q, Hu D. GAPDH is a Novel ferroptosis-related marker and correlates with Immune Microenvironment in Lung Adenocarcinoma. LID – 10.3390/metabo13020142 [doi] LID – 142. 10.3390/metabo13020142 PMC9961514 36837761

[60] Wang LA-O, Peng W, Wu T, Deng P, Zhao YL. Increased glutamine anabolism sensitizes non-small cell lung cancer to gefitinib treatment. 10.1038/s41420-018-0086-x PMC6085389 30109143

[61] Zhang H, Chen X, Wang J, Guang W, Han W, Zhang H, Tan X, Gu Y. EGR1 decreases the malignancy of human non-small cell lung carcinoma by regulating KRT18 expression. 10.1038/srep05416 PMC4080516 24990820

[62] Tanimura K, Yamada T, Horinaka M, Katayama Y, Fukui S, Morimoto K, Nakano T, Tokuda S, Morimoto Y, Iwasaku M et al. Inhibition of c-Jun N-terminal kinase signaling increased apoptosis and prevented the emergence of ALK-TKI-tolerant cells in ALK-rearranged non-small cell lung cancer. 10.1016/j.canlet.2021.09.018 34534615

[63] Min SA-O, Choe C, Roh SA-O. AQP3 increases intercellular cohesion in NSCLC A549 cell spheroids through exploratory cell protrusions. LID – 10.3390/ijms22084287 [doi] LID – 4287. 10.3390/ijms22084287 PMC8074759 33924231

[64] Wang S, Wu Y, Yang S, Liu X, Lu Y, Liu F, Li G, Tian GA-OX. miR-874 directly targets AQP3 to inhibit cell proliferation, mobility and EMT in non-small cell lung cancer. 10.1111/1759-7714.13428 PMC7262918 32301290

[65] Lin G, Chen L, Lin L, Lin H, Guo Z, Xu Y, Hu C, Fu J, Lin Q, Chen W et al. Comprehensive Analysis of Aquaporin Superfamily in Lung Adenocarcinoma. 10.3389/fmolb.2021.736367 PMC8542973 34708074

[66] Wang SA-O, Chen JJ, Jiang Y, Lei ZN, Ruan YA-O, Pan Y, Yam JA-OX, Wong MP. Xiao ZA-O. Targeting GSTP1 as therapeutic strategy against lung adenocarcinoma stemness and resistance to tyrosine kinase inhibitors. 10.1002/advs.202205262 PMC9982593 36709476

[67] Anichini AA-O, Perotti VE, Sgambelluri F, Mortarini R. Immune escape mechanisms in non small cell Lung Cancer. LID – 10.3390/cancers12123605 [doi] LID – 3605. 10.3390/cancers12123605 PMC7761620 33276569

[68] Lei X, Lei Y, Li JK, Du WX, Li RG, Yang J, Li J, Li F, Tan HB. Immune cells within the tumor microenvironment: Biological functions and roles in cancer immunotherapy. 10.1016/j.canlet.2019.11.009 31730903

[69] Dutta SA-O, Ganguly AA-O, Chatterjee KA-O, Spada SA-O, Mukherjee SA-OX. Targets of Immune escape mechanisms in Cancer: basis for Development and Evolution of Cancer Immune checkpoint inhibitors. LID – 10.3390/biology12020218 [doi] LID – 218. 10.3390/biology12020218 PMC9952779 36829496

[70] Wei Y, Vellanki RN, Coyaud É, Ignatchenko V, Li L, Krieger JR, Taylor P, Tong J, Pham NA, Liu G et al. CHCHD2 is coamplified with EGFR in NSCLC and regulates mitochondrial function and cell Migration. 10.1158/1541-7786.MCR-14-0165-T 25784717

[71] Yin X, Xia J, Sun Y, Zhang Z. CHCHD2 is a potential prognostic factor for NSCLC and is associated with HIF-1a expression. 10.1186/s12890-020-1079-0 PMC7020603 32054470

