# Supplementary figures and images for: Decoding temporal heterogeneity in NSCLC through machine learning and prognostic model construction
Source: World J Surg Oncol. 2024 Jun 13;22:156. doi: 10.1186/s12957-024-03435-0 (PMC11170806; doi:10.1186/s12957-024-03435-0)

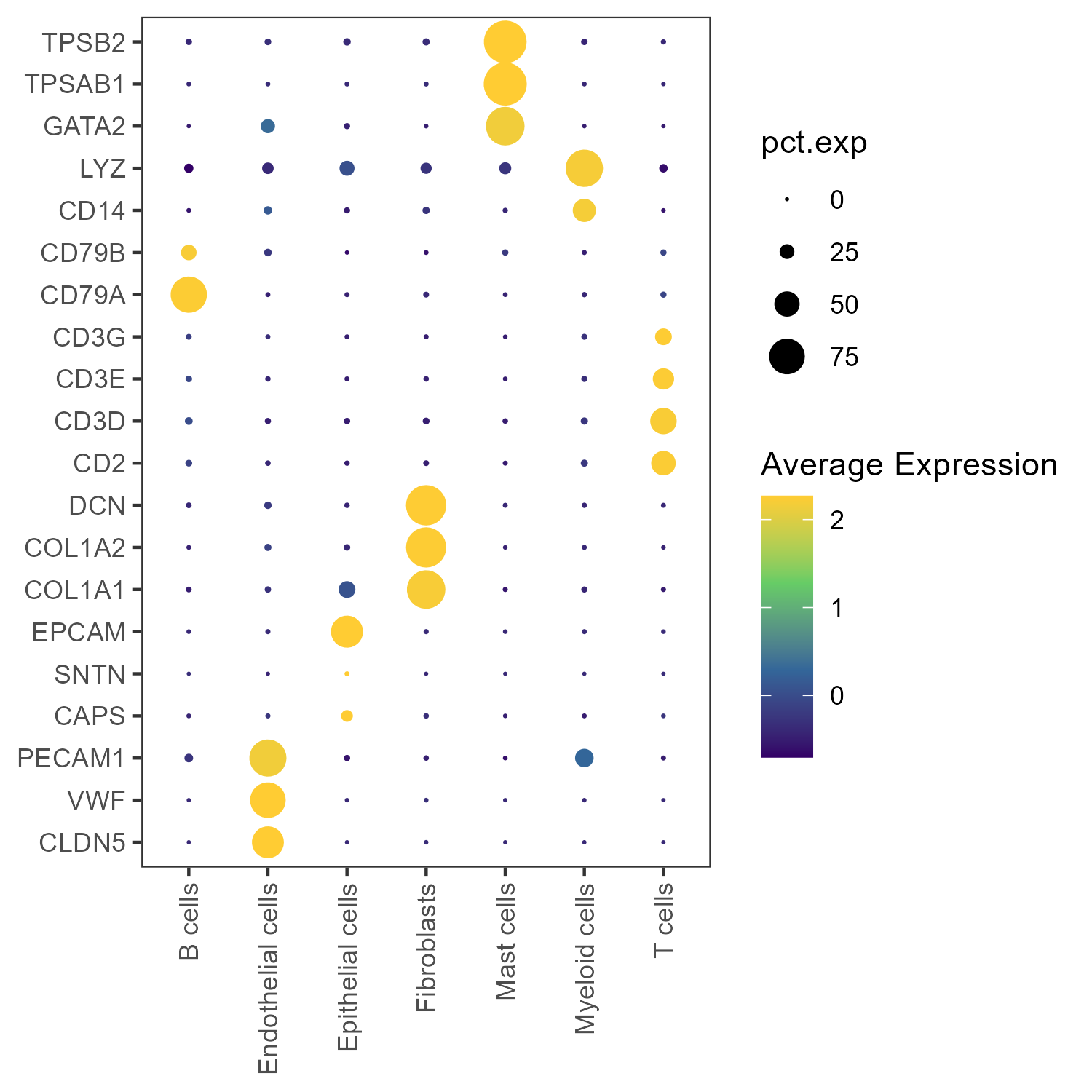

Supplement: Supplementary file 1 — Supplementary Material 1 [file 12957_2024_3435_MOESM1_ESM.png]
